# Prevalence of MRSA in Livestock, Including Cattle, Farm Animals, and Poultry, in Mainland China, Hong Kong Special Administrative Region, Sri Lanka, and Bangladesh: A Systematic Review and Meta-Analysis

**DOI:** 10.3390/microorganisms13040704

**Published:** 2025-03-21

**Authors:** Nilakshi Barua, Nannur Rahman, Martha C. F. Tin, Liuyue Yang, Abdul Alim, Farhana Akther, Nelum Handapangoda, Thamali Ayeshcharya Manathunga, Rasika N. Jinadasa, Veranja Liyanapathirana, Mingjing Luo, Margaret Ip

**Affiliations:** 1 Department of Microbiology, Faculty of Medicine, The Chinese University of Hong Kong, Prince of Wales Hospital, Shatin, New Territories, Hong Kong SAR, China; nilakshibarua@cuhk.edu.hk (N.B.); mnannuiu@link.cuhk.edu.hk (N.R.); bella.liuyuey@link.cuhk.edu.hk (L.Y.); 2 Department of Food Technology and Nutritional Science, Mawlana Bhashani Science and Technology University, Tangail 1902, Bangladesh; alim.food@mbstu.ac.bd (A.A.); farhana@mbstu.ac.bd (F.A.); 3 Faculty of Medical Sciences, University College London, London WC1E 6BT, UK; martha.tin.19@ucl.ac.uk; 4 Department of Microbiology, Faculty of Medicine, University of Peradeniya, Peradeniya 20400, Sri Lanka; nelumpamoda2@gmail.com (N.H.); veranja.liyanapathirana@med.pdn.ac.lk (V.L.); 5 Department of Veterinary Pathobiology, Faculty of Veterinary Medicine & Animal Science, University of Peradeniya, Peradeniya 20400, Sri Lanka; tmanathun2-c@my.cityu.edu.hk (T.A.M.); rnjinadasa@vet.pdn.ac.lk (R.N.J.); 6 CAS Key Laboratory of Quantitative Engineering Biology, Shenzhen Institute of Synthetic Biology, Shenzhen Institute of Advanced Technology, Chinese Academy of Sciences (CAS), Shenzhen 518055, China; mj.luo@siat.ac.cn; 7 Shenzhen Research Institute, The Chinese University of Hong Kong, Shenzhen 518057, China; 8 Centre for Gut Microbiota Research, Faculty of Medicine, The Chinese University of Hong Kong, Prince of Wales Hospital, Shatin, New Territories, Hong Kong SAR, China; 9 S. H. Ho Research Centre for Infectious Diseases, The Chinese University of Hong Kong, Shatin, New Territories, Hong Kong SAR, China

**Keywords:** MRSA, LA-MRSA, *Staphylococcus aureus*, Sri Lanka, Bangladesh, Mainland China, Hong Kong SAR

## Abstract

Methicillin-resistant *Staphylococcus aureus* (MRSA) can spread from animals to humans, but how it adapts to infect both is not fully understood. Our review aimed to determine the prevalence of MRSA in livestock, poultry, and companion animals in different countries, including Bangladesh, the Hong Kong SAR, Mainland China, and Sri Lanka. Articles were collected using PubMed, Embase, Web of Science, Scopus, CINAHL, and Google Scholar. Only prevalence studies that followed the PICO guidelines were included. A random-effects model meta-analysis was used to pool the data. The quality of the evidence and bias were assessed using the GRADEpro and Cochrane collaboration tools. Out of 1438 articles, 69 studies were eligible for meta-analysis. The studies showed significant heterogeneity (*I*^2^ = 97.00%, *p* < 0.0001) in the prevalence of MRSA colonization. Therefore, a random-effects model was used to determine the pooled prevalence of MRSA colonization, which was found to be 4.92% (95% CI: 3.79% to 6.18%). Begg’s test (*p* = 0.0002) and Egger’s test (*p* = 0.0044) revealed publication bias. Subgroup analysis of the pooled prevalence of MRSA showed a significant difference (*p* < 0.00001) when the subgroups were divided by country, MRSA detection method, whether pre-enrichment was performed or not, study period, sample collection location, and study population. Although significant factors can partially explain the heterogeneity, it is crucial to recognize the heterogeneity within different subgroups. The pooled prevalence of MRSA was found to vary significantly (*p* < 0.00001) among the study periods and has increased since the study period of 2020. Therefore, it is crucial to continuously monitor and implement measures to control the spread of MRSA in animals to minimize the risk of transmission to humans.

## 1. Introduction

Antimicrobial resistance (AMR) poses a crucial challenge to global health. The indiscriminate use of antibiotics undeniably contributes to the emergence of antimicrobial-resistant bacteria. This, in turn, facilitates the spread of antibiotic resistance genes and AMR bacteria among humans, domestic and farm animals, wildlife, and the environment [1]. Methicillin-resistant *Staphylococcus aureus* (MRSA) has been on the rise since it was first reported in the UK in 1961 [2]. MRSA is a pathogen of significant concern due to its resistance to multiple antibiotics. It can cause a range of infections, from minor skin infections to potentially fatal invasive infections. MRSA infections have become a global concern, affecting several regions and countries worldwide, such as Europe, Australia, the USA, and Asia. The frequency of MRSA cases varies by region, with Scandinavia reporting fewer cases and America (the United States, Canada, and Latin America) and Asia registering higher incidences [3]. The high morbidity and mortality rates associated with MRSA infections pose a significant threat to both human and veterinary healthcare.

The incidence of MRSA infections in livestock and companion animals has been on the rise. The colonization and spread of MRSA may be attributed to the injudicious use of antimicrobial drugs in animal husbandry and other agricultural activities, leading to the continuous exposure of humans and animals to antibiotics and inadequate infection control measures [4]. Farm animals and pets that carry antibiotic-resistant bacteria have the potential to transmit them to people and serve as a source of resistance genes that can move horizontally from commensal to pathogenic organisms present in a common microbial community and vice versa. The potential contribution of farm animals and pets to the spread of antimicrobial resistance among humans, given their significant levels of interaction with people and their environment, is a cause of concern and must be evaluated [5]. Molecular analysis has shown that, while some MRSA strains are host-specific, others can infect a broad range of hosts, including humans. An important example of this is ST398, which was initially isolated from pigs but has since been detected in several other companion and food-chain animals and humans [6].

Individuals having frequent contact with farm animals are at high risk of developing live-stock-associated MRSA (LA-MRSA) infections. In Germany, MRSA has been reported in farms raising turkeys, cattle, and pigs [7]. Studies indicate that 23–32% of Dutch pig farmers [8,9] and around 20% of North American pig farmers have acquired MRSA infections. MRSA is resistant to most antibiotics, which leads to longer hospital stays and an increased burden on healthcare and the economy [10,11]. New genetic variants of MRSA have been found in many sources, including food and animals, requiring further research. To further exacerbate these challenges, new antibiotics for MRSA are not being developed at a rapid pace [12,13]. A recent discovery of MRSA ST39 carrying *tsst-1* in Hong Kong food markets illustrates the unstoppable emergence of new MRSA lineages [14]. This suggests that livestock and other animals can serve as a long-term source of MRSA infections in humans.

As a result, it is important to pay close attention to managing farm animals and their potential impact on human health. It is crucial to be aware that farm animals and pets can carry staphylococci from other animals and even humans who are not part of the household. These new strains of bacteria can then be introduced into the home environment, which could lead to the re-colonization and re-infection of humans. Controlling the spread of staphylococci and maintaining the health and safety of both pets and humans are crucial [15], and one important step in this process is assessing the prevalence of MRSA in farm animals and pets.

Many countries face a high prevalence of antimicrobial resistance (AMR), exacerbated by the routine application of antibiotics in livestock for disease treatment and growth promotion. This situation necessitates a comprehensive regulatory framework to mitigate the risks associated with antibiotic misuse. The regulatory landscape of antibiotic use in animal healthcare significantly impacts the prevalence of MRSA in animals [16] and is characterized by significant challenges, including the overuse of antibiotics, a lack of awareness, and the inadequate enforcement of the existing regulations. In Bangladesh, The Department of Livestock Services (DLS) is responsible for implementing the National Action Plan (NAP) to combat AMR in the animal health sector [17]. The regulatory framework governing antibiotic use in animal healthcare in the Hong Kong Special Administrative Region is shaped by various policies implemented to combat antimicrobial resistance (AMR). The Hong Kong Strategy and Action Plan on Antimicrobial Resistance (HKSAP) 2017–2022 has been instrumental in addressing AMR through initiatives focused on education, surveillance, and stewardship. In addition, leading veterinarians rely on international standards for prescribing antibiotics [18]. The regulatory landscape of antibiotic use in animal healthcare in Mainland China has evolved significantly in response to the growing threat of antimicrobial resistance (AMR). The government has implemented various policies aimed at controlling antibiotic use in livestock, particularly in commercial farming. Key regulations include maximum residue levels for antibiotics, a list of permitted antibiotics, and guidelines for prescription-only use in animal production [19]. A comprehensive management system has been established, focusing on rational antibiotic use and AMR surveillance [20]. Sri Lanka has demonstrated its commitment to addressing AMR through its National Strategic Plan (NSP) for Combating Antimicrobial Resistance 2023–2028, which has a one health approach. The Veterinary Drug Control Authority under the purview of the Department of Animal Production and Health regulates veterinary antibiotic usage in the country. Encouragingly, the use of antibiotic growth promoters has been banned since 2018. Furthermore, veterinary products containing colistin and third- and fourth-generation cephalosporins have been discontinued. The importation of bulk pack antibiotic products weighing >1 kg/1 L intended for farm animal usage has also been banned, and veterinary antibiotic importers are now required to report the previous year’s sales volume to place new orders.

This study aimed to determine the prevalence of MRSA in livestock and poultry through a systematic review and meta-analysis according to the Preferred Reporting Items for Systematic Reviews and Meta-Analyses guidelines in countries in the Far East, including Bangladesh, Sri Lanka, the Hong Kong Special Administrative Region, and Mainland China [21], because the prevalence of MRSA is high in these countries [22]. We reviewed 1438 articles on the prevalence of MRSA in farm animals and companion animals from Bangladesh, China, the Hong Kong SAR, and Sri Lanka. Determining the degree of antibiotic resistance in MRSA present in the agricultural animals of these nations is also crucial. Our systematic review will aid social workers, healthcare providers, policymakers, and consumers in making more informed decisions and better managing and preparing for the illness burden [23,24]. Certain characteristics and potential issues, such as anthropozoonotic risks, can be identified by understanding the prevalence of MRSA, which can be further utilized in creating and implementing the control measures for MRSA within the framework of the one health perspective [25].

## 2. Methods

### 2.1. Protocol Design and Registration

The systematic review protocol was developed following the PRISMA-P 2015 and Cochrane systematic review guidelines [26,27]. The review protocol was registered in the International Prospective Register of Systematic Reviews (PROSPERO) in 2023 with the registration number CRD42023420188 [28]. The authors fully adhered to the protocol to accomplish this review and meta-analysis. The PRISMA checklist is available in Supplementary Table S1.

### 2.2. Search Strategies

The electronic bibliographic databases, such as PubMed (https://pubmed.ncbi.nlm.nih.gov/, accessed on 23 January 2024), SCOPUS (https://www.elsevier.com/products/scopus, accessed on 23 January 2024), Web of Science (SCI) (https://clarivate.com/academia-government/scientific-and-academic-research/research-discovery-and-referencing/web-of-science/, accessed on 23 January 2024), CINAHL (EBSCO) (https://www.ebsco.com/products/research-databases/cinahl-database, accessed on 23 January 2024), Embase (via Ovid) (https://www.elsevier.com/products/embase, accessed on 23 January 2024), and MEDLINE (via Ovid) (https://www.nlm.nih.gov/medline/medline_home.html, accessed on 23 January 2024), in addition to Google Scholar, were extensively searched to obtain material for this review and meta-analysis. When practical for our assessment, articles from Google or other databases were manually included. The same search techniques were used to collect data for each country. While there were no limitations on the publication period, we included only articles published in English and Chinese. The search string was “Methicillin-resistant *Staphylococcus aureus OR Staphylococcus aureus* OR Vancomycin-Resistant *Staphylococcus aureus* AND Livestock OR Domestic animals OR Cattle OR Poultry OR Cow OR Goat OR Sheep OR Chicken OR Buffalo OR Swine OR pigs AND Hong Kong OR Hong Kong SAR OR Hong Kong Special Administrative Region China OR Mainland China OR China OR Srilanka OR Sri Lanka OR Bangladesh”. The final database update took place in January 2024. The Supplementary Tables S2 and S3 contain comprehensive information on the search strategies and articles retrieved from several databases.

## 3. Eligibility for Study Selection

### 3.1. Inclusion Criteria

We followed a defined framework, PEO (P = population/subjects or problems, E = exposure, and O = outcomes), to determine the eligibility criteria of the articles [29]. Mainly observational studies, including cross-sectional, longitudinal, or cohort studies, were considered for eligibility. Only articles with full texts were considered for inclusion. The inclusion and exclusion criteria are detailed in Supplementary Table S4, which emerged throughout the title, abstract, and full-text screening.

### 3.2. Populations/Subjects

The subjects for the review were animals such as livestock, cattle, cows, goats, sheep, chickens, ducks, quails, turkeys, buffalo, swine, and companion animals. Only studies that mentioned a minimum of 30 individuals to collect samples were considered eligible for inclusion.

### 3.3. Exposure

This study aimed to characterize the exposure of farm animals or livestock to *Staphylococcus aureus* or MRSA. Therefore, studies that confirmed the colonization of *Staphylococcus aureus* or MRSA through culture, PCR, or other means were assessed for eligibility.

### 3.4. Outcomes of Interest

The main outcome of this review was determining the prevalence of MRSA in farm animals or livestock. Along with MRSA, the prevalence of *S. aureus* in farm animals and livestock and that of MRSA among the recovered *S. aureus* were set as the primary outcomes. Furthermore, any clinical manifestations brought on by MRSA or *S. aureus* exposure, important colonization risk factors, pathogen molecular traits, antibiotic resistance, etc., were regarded as secondary outcomes.

### 3.5. Exclusion Criteria

Because the databases were updated in January 2024, any research completed beyond that date was disregarded. Articles written in languages other than Chinese and English were likewise overlooked. News, letters, editorials, review pieces, brief communications, and gray literature were not accepted. We ignored data that had not been published before the end date. For collecting data, the authors were contacted directly if the manuscript had been accepted or had gone into pre-print. Studies were also disqualified if they included an MRSA investigation but failed to use reliable techniques to verify exposure or colonization. Additionally, papers with fewer than thirty subjects were disregarded.

### 3.6. Data Documentation

We recorded and documented the data from the included studies using a precisely designed data collection form (Supplementary Table S5). If necessary, the data were recorded from the figure when the data were not available in the text but in figures. One possible way to capture the missing data was to contact the authors personally. Only the initial test results for the same subject were used in the selected studies, involving multiple screenings for MRSA and *S. aureus*. In addition, the documentation of MRSA antibiotic resistance and molecular genetic characterization was performed. Furthermore, certain fundamental details were mentioned, such as the animal species, farm infrastructure, environment, and hygiene standards. To create the database, the following pieces of information were recorded: author names, publication year, study design, research or screening setting, nation or region of the study, sample sites, total number of target individuals, health status, *S. aureus* screening method, and MRSA identification method.

### 3.7. Quality Assessment and Reducing the Risk of Bias

COVIDENCE (www.covidence.org, accessed on 21 April 2024) was used to assess the eligibility of the articles and avoid potential bias risks. Six reviewers conducted abstract screening and full-text reviews with respect to each country and article inclusion for this review. The senior author resolved any disagreements. The Joanna Briggs Institution (JBI) checklist (https://jbi.global/sites/default/files/2020-08/Checklist_for_Prevalence_Studies.pdf, accessed on 31 May 2024) for studies reporting prevalence data was used to evaluate the bias risk in the selected publications [30]. Additionally, risk bias was evaluated using the RevMan 5.4 version of the Cochrane Collaboration tool (https://www.cochranelibrary.com/, accessed on 17 September 2024). Three categories were used to rate each potential bias: unknown, high risk, or low risk.

### 3.8. Data Analysis

Meta-analysis using Statistical Packages for the Social Science (SPSS), version 28, Chicago, IL, USA, RevMan 5.4, Cochrane Collaboration, London, UK, and MedCalc (MedCalc Software 22.2, Mariakerke, Belgium) was performed based on the primary (prevalence of MRSA and *S. aureus*) and secondary outcome data from the included studies. The Cochrane Collaboration criteria were used to interpret the heterogeneity among studies [27]. Values of *I*^2^ = 0–49%, *I*^2^ = 50–75%, and *I*^2^ > 75% were interpreted as low, moderate, and high degrees of heterogeneity, respectively [31,32]. Significant differences were determined using 95% confidence intervals. A funnel plot was employed to illustrate the asymmetry and publication biases of the studies. Regression of funnel asymmetry, rank correlation, Egger’s and Begg’s tests, and other methods were utilized to quantify the publication bias, because using a single test to measure it would have been unreliable [33,34].

## 4. Results

### 4.1. Characteristics of Studies and Quality Evaluation

**Figure 1:**
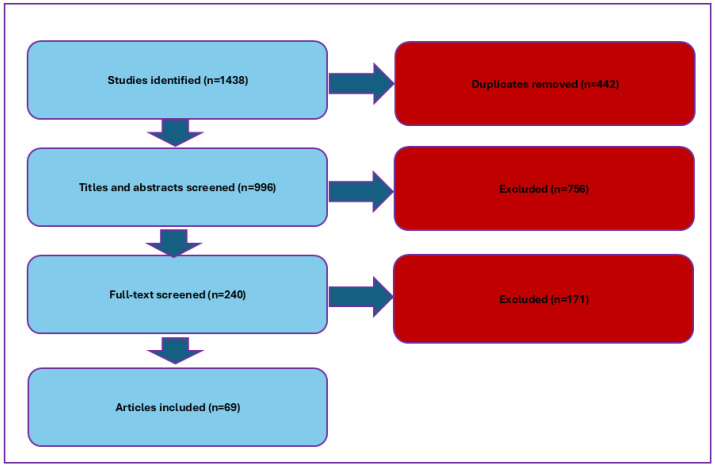
PRISMA flowchart outlining the study selection process.

A total of 1438 records were collected from various databases; the PRISMA flowchart in Figure 1 shows the study selection process. The 69 qualified studies used different methods for identifying MRSA. A total of 28 studies used the *mecA* gene [35,36,37,38,39,40,41,42,43,44,45,46,47,48,49,50,51,52,53,54,55,56,57,58,59,60,61,62], 22 used the cefoxitin disk diffusion method [63,64,65,66,67,68,69,70,71,72,73,74,75,76,77,78,79,80,81,82,83,84], 15 used the agar or broth dilution method [84,85,86,87,88,89,90,91,92,93,94,95,96,97,98], and only 2 used the selective agar plate method [99,100]. The remaining two did not mention the specific method [101,102]. All 69 studies were cross-sectional studies (Supplementary Table S6).

### 4.2. Meta-Analysis Results

#### 4.2.1. Overall Pooled Prevalence

**Figure 2:**
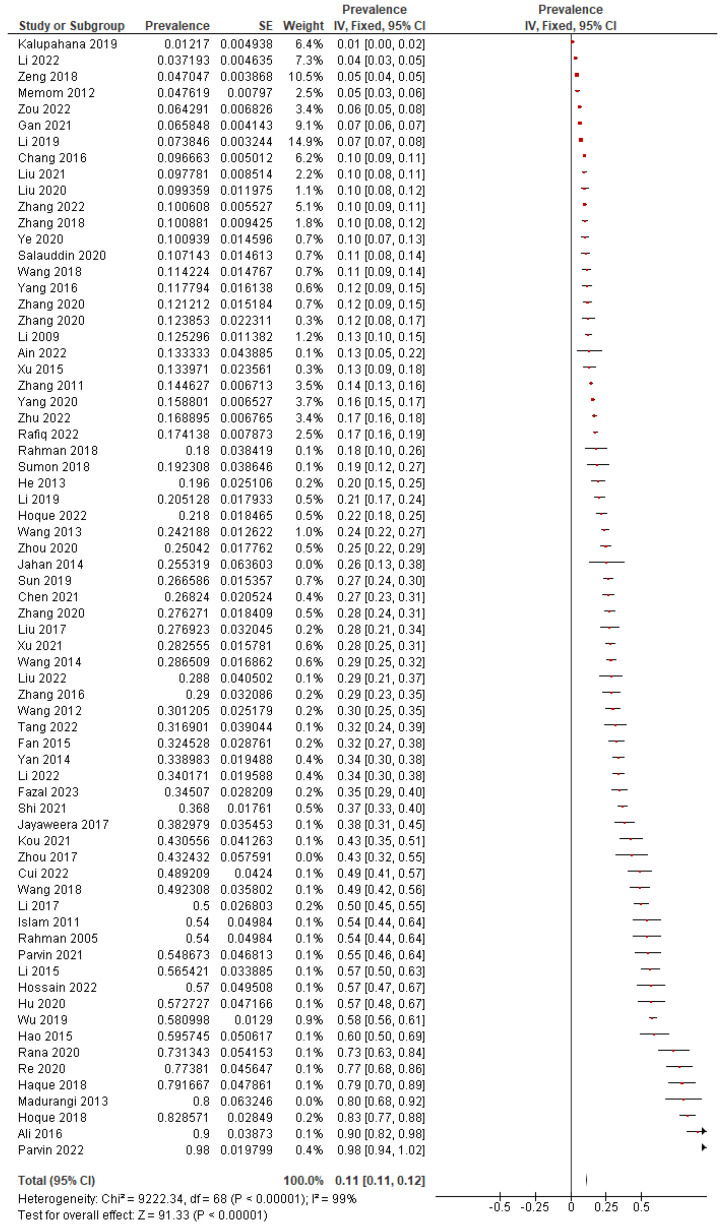
Forest plot for pooled *S. aureus* prevalence and 95% CI for all selected studies [35,36,37,38,39,40,41,42,43,44,45,46,47,48,49,50,51,52,53,54,55,56,57,58,59,60,61,62,63,64,65,66,67,68,69,70,71,72,73,74,75,76,77,78,79,80,81,82,83,84,85,86,87,88,89,90,91,92,93,94,95,96,97,98,99,100,101,102,103,104].

In the 69 studies included in the meta-analysis, 9956 *S. aureus* (SA) strains were detected from 58361 samples. A high level of heterogeneity (*I*^2^ = 99%, *p <* 0.0001) was observed; therefore, a random-effects model was established to determine the pooled prevalence of *S. aureus* (SA) among the population (28.46%; 95% confidence interval (CI): 24.80% to 32.26%) (Figure 2). Begg’s test (*p* = 0.0001) and Egger’s test (*p* < 0.0001) were conducted to assess the publication bias of the literature. The publication bias regarding the SA numbers may be due to the smaller sample sizes in a few of the included studies. This means that studies with smaller sample sizes may show lower numbers of SA cases, which could potentially skew the overall results.

**Figure 3:**
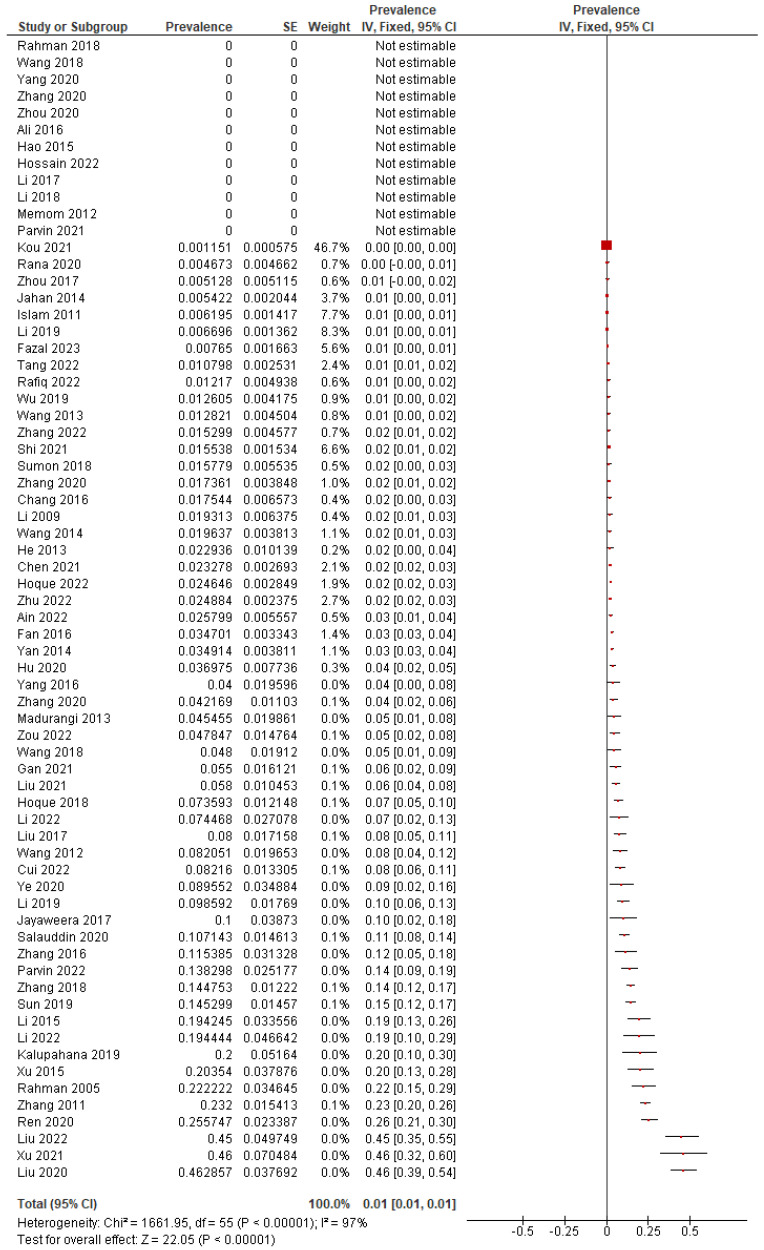
Forest plot for pooled MRSA prevalence and 95% CI for all selected studies [35,36,37,38,39,40,41,42,43,44,45,46,47,48,49,50,51,52,53,54,55,56,57,58,59,60,61,62,63,64,65,66,67,68,69,70,71,72,73,74,75,76,77,78,79,80,81,82,83,84,85,86,87,88,89,90,91,92,93,94,95,96,97,98,99,100,101,102,103,104].

A total of 1921 MRSA cases were detected in the included articles; the total prevalence of MRSA ranged from 0% to 46.29%. The analysis of 69 studies showed significant heterogeneity (*I*^2^ = 97.00%, *p* < 0.0001) in the prevalence of MRSA colonization (Figure 3). The pooled prevalence of MRSA was found to be 4.92% (95% CI: 3.79% to 6.18%) using a random-effects method, indicating that a considerable proportion of the population carries MRSA. Begg’s (*p* = 0.0002) and Egger’s tests (*p* = 0.0044) revealed publication bias.

#### 4.2.2. Subgroup Analyses

Six independent factors were used for the subgroup analyses for the prevalence of MRSA, as shown in Table 1. The pooled prevalence of MRSA differed significantly when the subgroup was divided country-wise (*p* < 0.00001): Bangladesh, Mainland China, the Hong Kong SAR, and Sri Lanka. The pooled prevalence of MRSA determined using the random-effects model in Bangladesh was 12.29% (95% CI: 7.09 to 18.67; *I*^2^ = 96.00%), that of Mainland China and Hong Kong was 4.65% (95% CI: 3.59 to 5.86; *I*^2^ = 97.00%), and that of Sri Lanka was 3.83% (95% CI: 0.003 to 15.09; *I*^2^ = 96.00%). The pooled prevalence of MRSA differed significantly based on the detection method (*p* < 0.00001). The pooled prevalence of MRSA determined using the random-effects model with broth microdilution or agar dilution was 1.67% (95% CI: 0.96 to 2.56; *I*^2^ = 88.00%), that determined through PCR of the *mecA* and/or *mecC* genes was 5.20% (95% CI: 3.58 to 7.11; *I*^2^ = 97.00%), that determined through disk diffusion was 5.54% (95% CI: 3.18 to 8.51; *I*^2^ = 99.00%), and that determined using selective media was 5.56% (95% CI: 0.41 to 26.47; *I*^2^ = 99.00%). The subgroup analysis of the pooled MRSA prevalence differed significantly (*p* < 0.00001) depending upon whether pre-enrichment (i.e., broth enrichment for improved detection sensitivity) was performed or not; it was 5.41% (95% CI: 3.94 to 7.11; *I*^2^ = 97.00%) if it was performed and 2.54% (95% CI: 1.61 to 3.68; *I*^2^ = 72.00%) if not, before *S. aureus* detection. The pooled prevalence of MRSA also differed significantly (*p <* 0.0001) based on the study period. The pooled MRSA prevalence for the studies conducted between 2010 and 2019 was 3.62% (95% CI: 2.31 to 5.20; *I*^2^ = 97.00%), and that for those conducted between 2020 and 2023 was 6.25% (95% CI: 4.45 to 8.32; *I*^2^ = 97.00%). Based on the location of sample collection, subgroup analysis showed a significant difference (*p <* 0.0001) in the pooled prevalence of MRSA. The prevalence was 4.15% (95% CI: 2.88 to 5.63; *I*^2^ = 99.00%) when samples were collected from urban locations and 9.49% (95% CI: 4.49 to 16.10; *I*^2^ = 98.00%) when they were from non-urban locations. The subgroup analysis (Table 2) based on the study population showed that the pooled MRSA prevalence differed significantly (*p <* 0.0001). The prevalence in cats was 0.11% (95% CI: 0.005 to 0.36), that in chickens was 3.05% (95% CI: 1.12 to 5.88; *I*^2^ = 95.00%), that in cows was 1.91% (95% CI: 0.56 to 4.05; *I*^2^ = 75.00%), that in dogs was 4.98% (95% CI: 0.0004 to 19.07; *I*^2^ = 70.00%), that in ducks was 1.26% (95% CI: 0.45 to 2.48; *I*^2^ = 53.00%), that in goats was 2.60% (95% CI: 0.16 to 7.87; *I*^2^ = 9.00%), that in pigs was 5.96% (95% CI: 3.59 to 8.87; *I*^2^ = 96.00%), that in raw milk was 4.23% (95% CI: 2.30 to 6.73; *I*^2^ = 99.00%), that in yaks was 1.25% (95% CI: 0.10 to 3.65; *I*^2^ = 65.00%), that in beef was 2.20% (95% CI: 0.20 to 6.28; *I*^2^ = 96.00%), that in chicken meat was 8.42% (95% CI: 3.15 to 15.92; *I*^2^ = 93.00%), and that in pork was 3.94% (95% CI: 1.26 to 8.02; *I*^2^ = 89.00%).

#### 4.2.3. Risk of Bias Assessment

**Figure 4:**
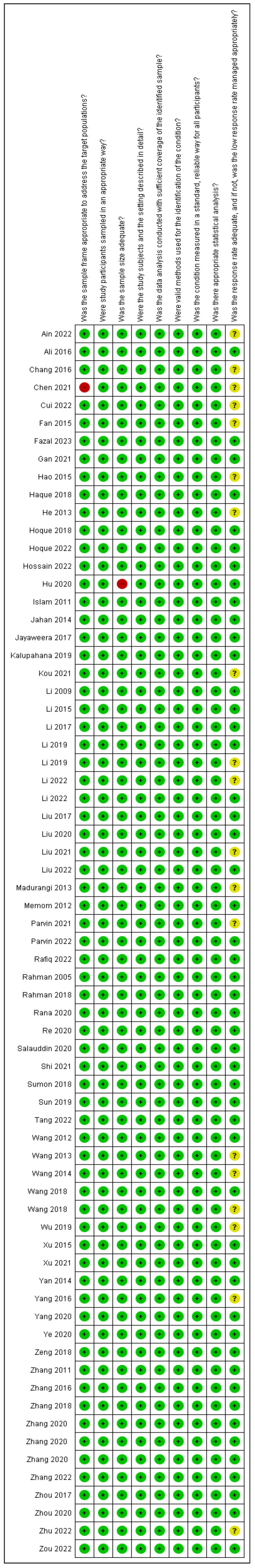
Quality assessment of the risk of bias for the included MRSA prevalence studies [35,36,37,38,39,40,41,42,43,44,45,46,47,48,49,50,51,52,53,54,55,56,57,58,59,60,61,62,63,64,65,66,67,68,69,70,71,72,73,74,75,76,77,78,79,80,81,82,83,84,85,86,87,88,89,90,91,92,93,94,95,96,97,98,99,100,101,102,103,104].

**Figure 5:**
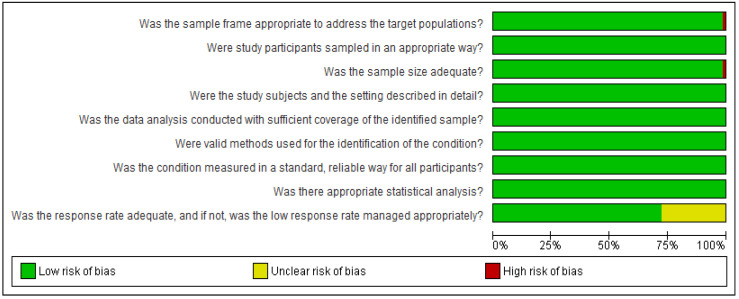
Summary graph of the risk of bias for MRSA prevalence studies.

Figure 4 displays the outcomes of the quality evaluation conducted on pertinent studies that report the prevalence of MRSA and SA in animals. Of the 69 prevalence studies, 48 were rated as excellent in terms of bias elements. Except for one study, Chen 2021 [85], all had appropriate sampling frames within the relevant regions for accurately calculating the prevalence. Each study provided unambiguous information regarding the number of samples analyzed and the number of MRSA samples detected. The prevalence studies showed minimal coverage bias, utilizing a consistent sample size for each unique subgroup. The summary graph indicated that the performance and detection biases were less than 25% (Figure 5).

#### 4.2.4. Publication Bias

**Figure 6:**
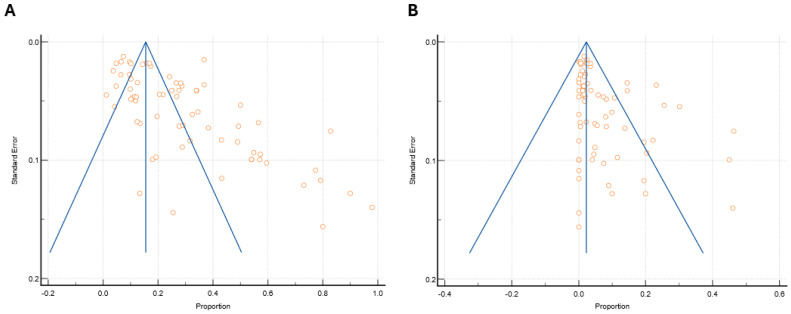
Funnel plot to detect the presence of publication bias, with effect sizes plotted on the x-axis and standard errors on the y-axis in (**A**) *S. aureus* and (**B**) MRSA of all selected studies.

The phenomenon of publication bias arises when the outcomes of a study have an impact on its likelihood of being published. Typically, studies with larger sample sizes and more significant effects tend to be published more frequently than smaller studies with weaker effects. This creates a publication bias. Funnel plots were utilized to detect the presence of publication bias, with effect sizes plotted on the x-axis and standard errors on the y-axis, as depicted in Figure 6.

Upon visual examination, it was observed that the studies showed an asymmetrical distribution on either side.

This asymmetrical pattern indicates that the likelihood of publication bias is high. Egger’s regression test (Supplementary Table S7) was utilized to quantify the presence of asymmetry to eliminate any subjective interpretations from the funnel plot visualizations. The results of Egger’s regression test showed significant publication bias for both SA and MRSA, with an intercept of 9.18 (95% Cl: 5.64 to 12.72), *p* ≤ 0.0001, for prevalence and with an intercept of 4.89 (95% CI: 2.43 to 7.37), *p* = 0.004 respectively.

#### 4.2.5. Quality of Evidence According to GRADEpro Analysis

The quality of the evidence of the studies was analyzed using the Cochrane review tool GRADEpro (https://www.gradepro.org/accessed on 15 October 2024), with respect to the prevalence of MRSA in livestock and pet animals. Table 3 shows that the certainty for the overall prevalence of MRSA was high (⨁⨁⨁⨁), revealing that the prevalence of MRSA in livestock and companion animals was high. However, the certainty varied from low to high in the subgroup analysis. This may be attributed to the fact that different types of studies were included in this review, and the prevalence rates among different animals are not the same. The settings of the respective studies were also different, resulting in high heterogeneity among the studies. The detection methods, farm locations, and enrichment of the samples also differed among the studies, which may have contributed to the inconsistency observed in the certainty assessment of the subgroup analysis. In addition, some of the studies included very few samples for assessing MRSA prevalence, and the study sample size was not calculated statistically.

### 4.3. Antibiotic Resistance Characterization

The antibiotic resistance traits of MRSA isolates were analyzed in 69 studies, out of which 37 studies were considered (Supplementary Table S8). High pooled resistance rates (i.e., higher than 60%) were observed for 18 antibiotics, with the highest being for ampicillin (99.6%; *I*^2^ *=* 0.00%), penicillin (98.8%; *I*^2^ = 41.92%), and oxacillin (98.8%; *I*^2^ = 19.25%). However, the rates of resistance to some antibiotics, such as nitrofurantoin (7.6%; *I*^2^ = 90.50%), quinupristin/dalfopristin (8.8%; *I*^2^ = 72.55%), and rifampin (12.7%; *I*^2^ = 90.90%), remained low. Only one isolate was found by Wang et al. (2018) [50] to be resistant to enrofloxacin, one isolate was found by Wu et al. (2019) [73] to be resistant to teicoplanin, and Liu et al. (2021) [46] found fifteen isolates that were resistant to vancomycin.

### 4.4. Molecular Genetic Characterization

Only a few studies have characterized the molecular genetic features of MRSA strains in their target population. Among the 11 [38,46,72,79,80,81,89,91,99,100,103] studies, the spa-typing of 454 isolates revealed that spa type t899 (281/454) was predominant, followed by t011 (30/454), t2616 (22/454), t437 (21/454), t571 (21/454), and t034 (19/454). The spa types t002 (3/454), t7400 (2/454), t3521 (2/454), t189 (2/454), t5390 (1/454), t524 (1/454), t127 (1/454), t2592 (2/454), t5762 (1/454), t4403 (1/454), t304 (1/454), and t10774(2/454) were not as common or prevalent. The staphylococcal cassette chromosome *mec* encoding methicillin resistance (SCC*mec*) type was characterized in only nine studies [38,72,80,85,87,89,90,91,99]; type IV (69/145) was observed to be the most prevalent SCCmec type, followed by type III (32/145), type V (30/145), type I (5/145), type XII (3/145), and type II (2/145). The SCC*mec* types of four isolates (4/145) were not identifiable. The multilocus sequence typing (MLST) of 409 isolates from 18 studies [37,38,50,69,72,73,75,80,81,85,89,91,94,95,98,99,103] revealed that ST9 was the most predominant ST type (156/409), followed by ST59 (92/409), ST398 (77/409), ST239 (19/409), ST1 (11/409), ST88 (8/409), ST5 (7/409), and ST338 (6/409). The ST types ST7 and ST630 had 5/409 each, followed by ST188 (4/409) and ST6 (3/409), while ST45 and ST4513 had 2/403 each. ST22, ST10, ST71, ST72, ST335, ST1376, ST943, ST968, ST3239, and ST3304 had 1/409 each.

## 5. Discussion

Our study estimated the prevalence of MRSA in animals using 69 studies published between 2010 and 2022. The pooled prevalences of *S. aureus* and MRSA in farm animals were 28.46% (95% CI: 24.80% to 32.26%) and 4.92% (95% CI: 3.79% to 6.18%), respectively. Our findings showed that the prevalence (95% CI) of MRSA was highest in chicken meat, with a rate of 8.42% (3.15–15.92) among all the animals studied. It is worth noting that the pooled prevalence of MRSA was found to be higher than the 8% (5% to 11%) reported in a previous study by Ribeiro et al., a systematic review and meta-analysis to assess the prevalence of MRSA in poultry and poultry meat worldwide [105]. The study also conducted a subgroup analysis based on the study period, which showed an increase in the MRSA prevalence from 3.9% (95% CI: 2.3–6.1) in 2010–2019 to 5.8% (95% CI: 3.8–8.3) in 2020–2023. The increase in MRSA in companion and farm animals has become a cause for concern. The emergence of antimicrobial-resistant microorganisms poses a significant threat to public health, food security, and animal welfare. Therefore, there is an urgent need for stronger measures to control the spread of these resistant bacteria in the farm environment and improve biosecurity practices to prevent their transmission to humans. The implementation of more stringent regulations and guidelines for the use of antibiotics in animal husbandry, as well as the increased surveillance and monitoring of resistant strains, can help to safeguard both animal and human populations from the growing threat of antimicrobial resistance. Therefore, the results of this study highlight the need for the continued monitoring and implementation of measures to control the spread of MRSA in animals and minimize the risk of transmission to humans.

It is important to consider that there are some limitations to the meta-analysis conducted. Firstly, the restriction on the languages of the included publications to English and Chinese may have introduced bias to the prevalence estimates. Secondly, the inclusion of studies with substantial heterogeneity and publication bias may have affected the accuracy of the results. However, it should be noted that, given the small sample size of 69 studies, the *I*^2^ estimate may not be entirely precise. Furthermore, Shi et al. (2021) [71] provided a significant fraction of the isolates (174 isolates) in our analysis, which could have led to systematic mistakes in determining the rate of antibiotic resistance and in genetic characterization. Thirdly, the potential for underestimating the prevalence of MRSA colonization in a meta-analysis is a concern, due to the inclusion of studies that employed culture-based methods with lower sensitivity in lieu of the PCR method to detect *S. aureus*. This discrepancy could have resulted in an underestimation of MRSA colonization rates and potentially impacted the accuracy of the meta-analysis. Therefore, it is crucial to consider the limitations of the methodology employed in the studies included in the meta-analysis and exercise discretion when interpreting the findings. Finally, given the significant variation in MRSA prevalence across different regions, conducting more cross-regional routine surveillance studies is imperative. Such studies will not only enable us to understand the current infection landscape in various regions, but also help us to identify the key factors contributing to the prevalence of MRSA in these areas. It is worth noting that the number of studies conducted tends to be higher in regions with higher reported infection cases. Therefore, conducting more studies across different regions is essential to ensure a more balanced and comprehensive understanding of the MRSA landscape.

## 6. Conclusions

Our research found that the prevalence of MRSA in Bangladesh was higher than that in the other regions included in the study. A subgroup analysis of the overall MRSA prevalence indicated notable differences (*p* < 0.00001) based on several factors, such as the country, detection method, pre-enrichment status, study period, sample location, and population studied. While these significant factors help to explain some of the variability, it is essential to acknowledge the heterogeneity present within the various subgroups. Our findings provide valuable insights into the prevalence and antibiotic resistance patterns of MRSA isolates from farm animals and can aid in the development of effective treatment strategies. Healthcare professionals should closely monitor MRSA and customize decolonization as well as antibiotic treatment plans based on the specific resistance patterns of the isolated strains. Further research is warranted to identify new antibiotics and alternative treatment options for MRSA infections. Further empirical studies are required to assess the prevalence of MRSA in livestock and its potential implications for human health in both the short and long term. Extensive surveillance studies across various regions and settings, including hospitals, farms, and food supply chains, will aid us in better understanding the prevalence and transmission dynamics of MRSA in both human and animal populations. This should include the use of standardized detection methods to ensure the comparability of results across studies. Investigations of the genetic mechanisms underlying antibiotic resistance in MRSA strains that are isolated from different sources, including livestock and food products, are required to identify potential targets for new treatment strategies and to inform public health interventions aimed at reducing the spread of resistant strains.

## Figures and Tables

**Table 1:** The subgroup analysis focused on the prevalence of MRSA in livestock, poultry, and companion animals.

	Number of Studies	MRSA Prevalence % (95% CI)	* I^2^%	*p*-Value (Between-Group)
Country				<0.00001
Bangladesh	17	12.29 (7.09 to 18.67)	96.00%	
China and HK	49	4.65 (3.59 to 5.86)	9.00%	
Sri Lanka	3	3.83 (0.003 to 15.09)	96.00%	
	69			
Detection method for MRSA				<0.00001
Broth microdilution or agar dilution	15	1.67 (0.96 to 2.56)	88.00%	
*mecA* and/or *mecC* PCR	28	5.20 (3.58 to 7.11)	97.00%	
Disk diffusion	22	5.54 (3.18 to 8.51)	96.00%	
Selective media	2	5.56 (0.41 to 26.47)	99.00%	
No method mentioned (excluded from subgroup analysis)	2	---	---	
	69			
Pre-enrichment before *S. aureus* detection				<0.00001
Yes	53	5.41 (3.94 to 7.11)	97.00%	
No	7	2.54 (1.61 to 3.68)	72.00%	
Not mentioned (excluded from subgroup analysis)	9	---	---	
	69			
Study period				<0.00001
2000–2009	2	1.19 (0.67 to 8.74)	** Not applicable	
2010–2019	35	3.62 (2.31 to 5.20)	97.00%	
2020–now	32	6.25 (4.45 to 8.32)	97.00%	
	69			
Study location				<0.00001
Urban	40	4.15 (2.88 to 5.63)	99.00%	
Rural	11	9.49 (4.49 to 16.10)	98.00%	
Not mentioned (excluded from subgroup analysis)	18	--	--	
	69			

* The *I*^2^ values were categorized to indicate levels of heterogeneity: low (*I*^2^ = 0–49%), moderate (*I*^2^ = 50–75%), and high (*I*^2^ > 75%). A significance level of 5% (*p* < 0.05) was used to determine the significant differences between treatment efficacies. ** Not applicable, as the prevalence of MRSA in Li et al., 2009 [66] is zero.

**Table 2:** The subgroup analysis focused on the prevalence of MRSA in livestock, poultry, and companion animals based on the study population.

Study Population		MRSA Prevalence % (95% CI)	* I^2^%	*p*-Value (Between-Group)
				<0.00001
Buffalos (Excluded from subgroup analysis)	1	--	--	
Cats	2	0.11 (0.005 to 0.36)	** Not applicable	
Chickens	10	3.05 (1.12 to 5.88)	95.00%	
Cows	11	1.91 (0.56 to 4.05)	75.00%	
Dogs	2	4.98 (0.0004 to 19.07)	70.00%	
Ducks	3	1.26 (0.45 to 2.48)	53.00%	
Goats	4	2.60 (0.16 to 7.87)	9.00%	
Pigs	19	5.96 (3.59 to 8.87)	96.00%	
Raw Milk	26	4.23 (2.30 to 6.73)	99.00%	
Sheep (Excluded from subgroup analysis)	1	--	--	
Yaks	2	1.25 (0.10 to 3.65)	65.00%	
Beef	4	2.20 (0.20 to 6.28)	96.00%	
Chicken meat	8	8.42 (3.15 to 15.92)	93.00%	
Duck meat (Excluded from subgroup analysis)	1	--	--	
Pork	8	3.94 (1.26 to 8.02)	89.00%	

* The *I*^2^ values were categorized to indicate levels of heterogeneity: low (*I*^2^ = 0–49%), moderate (*I*^2^ = 50–75%), and high (*I*^2^ > 75%). A significance level of 5% (*p* < 0.05) was used to determine the significant differences between treatment efficacies. ** Not applicable, as the prevalence of MRSA in Rahman et al., 2018 [48] is zero.

**Table 3:** GRADEpro summary of findings of prevalence of MRSA in livestock animals and companion animals for evaluating the quality of evidence assessed by two independent reviewers.

Certainty Assessment							Effect			Certainty	Importance
**No. of Studies**	Study Design	Risk of Bias	Inconsistency	Indirectness	Imprecision	Other Considerations	No. of Events	Total Samples	Relative Ratio (95% * CI)		
Prevalence of MRSA among livestock, including poultry, cattle, pigs, and companion animals (follow-up: range 6 weeks to 8 weeks; assessed with prevalence, scale from 0 to 1)											
68	Non-randomized studies	Not serious	Serious	Not serious	Serious	Strong association, all plausible residual confounding evidence would reduce the demonstrated effect	1766	55,417	0.03 (0.01 to 0.02)	⨁⨁⨁⨁ High	Critical
Prevalence of MRSA among livestock according to detection method; broth microdilution or agar dilution (follow-up: range 4 weeks to 6 weeks; assessed with prevalence; scale from 0 to 1)											
15	Non-randomized studies	Not serious	Serious	Not serious	Not serious	None	209	19,442	0.01 (001 to 0.01)	⨁⨁⨁◯ Moderate	Important
Prevalence of MRSA among livestock according to detection method; mecA or mecC (follow-up: range 6 weeks to 8 weeks; assessed with prevalence; scale from 0 to 1)											
28	Non-randomized studies	Not serious	Serious	Not serious	Serious	None	775	25,151	0.03 (0.01 to 0.01)	⨁⨁◯◯ Low	Important
Prevalence of MRSA among livestock according to detection method; disk diffusion (follow-up: range 6 weeks to 8 weeks; assessed with prevalence, scale from 0 to 1)											
22	Non-randomized studies	Not serious	Serious	Not serious	Not serious	None	617	12,824	0.05 (0.02 to 0.05)	⨁⨁⨁◯ Moderate	Important
Prevalence of MRSA among livestock in the pre-enrichment samples (follow-up: range 6 weeks to 8 weeks; assessed with prevalence, scale from 0 to 1)											
53	Non-randomized studies	Not serious	Serious	Not serious	Serious	Publication bias suspected, strong association, all plausible residual confounding evidence would reduce the demonstrated effect	1283	39,599	0.03 (0.01 to 0.02)	⨁⨁⨁◯ Moderate	Important
Prevalence of MRSA among livestock in the samples without pre-enrichment (follow-up: range 6 weeks to 8 weeks; assessed with prevalence, scale from 0 to 1)											
7	Non-randomized studies	Not serious	Serious	Not serious	Not serious	Strong association, all plausible residual confounding evidence would reduce the demonstrated effect	123	5327	0.02 (0.02 to 0.02)	⨁⨁⨁⨁ High	Important
Prevalence of MRSA among livestock based on farm location; urban farm (follow-up: range 4 weeks to 6 weeks; assessed with prevalence, scale from 0 to 1)											
40	Non-randomized studies	Not serious	Serious	Not serious	Serious	None	834	27,540	0.03 (0.02 to 0.025)	⨁⨁◯◯ Low	Important
Prevalence of MRSA among livestock based on farm location; rural farm (follow-up: range 4 weeks to 8 weeks; assessed with prevalence, scale from 0 to 1)											
10	Non-randomized studies	Not serious	Not serious	Not serious	Not serious	None	405	10,722	0.037 (0.01 to 0.02)	⨁⨁⨁⨁ High	Important

* CI: confidence interval.

## Data Availability

The original contributions presented in this study are included in the article/Supplementary Materials. Further inquiries can be directed to the corresponding author.

## Supplementary Materials

The following supporting information can be downloaded at: https://www.mdpi.com/article/10.3390/microorganisms13040704/s1. Table S1: PRISMA Checklist; Table S2: Search strategies; Table S3: An example of search results from PubMed for different countries, including Hong Kong, China, Sri Lanka, and Bangladesh.; Table S4: Study inclusion and exclusion criteria.; Table S5: Data collection form; Table S6: Characteristics of the studies; Table S7: Publication bias summary; Table S8: Antibiotic resistance characterisation.
