# Supplementary material for: Prevalence of MRSA in Livestock, Including Cattle, Farm Animals, and Poultry, in Mainland China, Hong Kong Special Administrative Region, Sri Lanka, and Bangladesh: A Systematic Review and Meta-Analysis
Source: Microorganisms. 2025 Mar 21;13(4):704. doi: 10.3390/microorganisms13040704 (PMC12029740; doi:10.3390/microorganisms13040704)
Supplement: Supplementary file 1 [file microorganisms-13-00704-s001.zip › microorganisms-3466034-supplementary.pdf]

Supplementary Table S1

PRISMA Checklist

Section/topic	#	Checklist item	Reported on page #
**TITLE**			
Title	1	Identify the report as a systematic review, meta-analysis, or both.	1
**ABSTRACT**			
Structured summary	2	Provide a structured summary including, as applicable: background; objectives; data sources; study eligibility criteria, participants, and interventions; study appraisal and synthesis methods; results; limitations; conclusions and implications of key findings; systematic review registration number.	1
**INTRODUCTION**			
Rationale	3	Describe the rationale for the review in the context of what is already known.	3-6
Objectives	4	Provide an explicit statement of questions being addressed with reference to participants, interventions, comparisons, outcomes, and study design (PICOS).	6-9, Table 1
**METHODS**			
Protocol and registration	5	Indicate if a review protocol exists, if and where it can be accessed (e.g., Web address), and, if available, provide registration information, including registration number.	6,
Eligibility criteria	6	Specify study characteristics (e.g., prevalence) and report characteristics (e.g., years considered, language, publication status) used as criteria for eligibility, giving rationale.	6-9, Table 1
Information sources	7	Describe all information sources (e.g., databases with dates of coverage, contact with study authors to identify additional studies) in the search and date last searched.	6-7
Search	8	Present full electronic search strategy for at least one database, including any limits used, such that it could be repeated.	Supplementary Table 2 and Supplementary Table 3
Study selection	9	State the process for selecting studies (i.e., screening, eligibility, included in systematic review, and, if applicable, included in the meta-analysis).	6-9Table 1,
Data collection process	10	Describe method of data extraction from reports (e.g., piloted forms, independently, in duplicate) and any processes for obtaining and confirming data from investigators.	9, Supplementary Table 4
Data items	11	List and define all variables for which data were sought (e.g., PICOS, funding sources) and any assumptions and simplifications made.	7-9, 11, Table 1
Risk of bias in individual studies	12	Describe methods used for assessing risk of bias of individual studies (including specification of whether this was done at the study or outcome level), and how this information is to be used in any data synthesis.	9-10
Summary measures	13	State the principal summary measures (e.g., risk ratio, difference in means).	

Section/topic	#	Checklist item	Reported on page #
Synthesis of results	14	Describe the methods of handling data and combining results of studies, if done, including measures of consistency (e.g., I^2) for each meta-analysis.	10
Risk of bias across studies	15	Specify any assessment of risk of bias that may affect the cumulative evidence (e.g., publication bias, selective reporting within studies).	10
Additional analyses	16	Describe methods of additional analyses (e.g., sensitivity or subgroup analyses, meta-regression), if done, indicating which were pre-specified.	10
**RESULTS**			
Study selection	17	Give numbers of studies screened, assessed for eligibility, and included in the review, with reasons for exclusions at each stage, ideally with a flow diagram.	11, Figure 1
Study characteristics	18	For each study, present characteristics for which data were extracted (e.g., study size, PICOS, follow-up period) and provide the citations.	11-15, Supplementary Table 5
Risk of bias within studies	19	Present data on risk of bias of each study and, if available, any outcome level assessment (see item 12).	18-19, Figure 4 and 5
Results of individual studies	20	For all outcomes considered (benefits or harms), present, for each study: (a) simple summary data for each intervention group (b) effect estimates and confidence intervals, ideally with a forest plot.	12-18, Figure 2, Figure3
Synthesis of results	21	Present results of each meta-analysis done, including confidence intervals and measures of consistency.	10-11, Figure 2 and 3, Table 2, Table 3
Risk of bias across studies	22	Present results of any assessment of risk of bias across studies (see Item 15).	18-20,22, Figure 4, Figure 5, Figure 6, Table 4
Additional analysis	23	Give results of additional analyses, if done (e.g., sensitivity or subgroup analyses, meta-regression [see Item 16]).	15-18, 21-24, Table 2, Table 3
**DISCUSSION**			
Summary of evidence	24	Summarize the main findings including the strength of evidence for each main outcome; consider their relevance to key groups (e.g., healthcare providers, users, and policy makers).	24-25
Limitations	25	Discuss limitations at study and outcome level (e.g., risk of bias), and at review-level (e.g., incomplete retrieval of identified research, reporting bias).	24-25
Conclusions	26	Provide a general interpretation of the results in the context of other evidence, and implications for future research.	25
**FUNDING**			
Funding	27	Describe funding sources for the systematic review and other support (e.g., supply of data); role of funders for the systematic review.	25

Supplementary Table S2: Search strategies

Country	Search strategies
**Hong Kong**	("Methicillin-Resistant Staphylococcus aureus"[MeSH Terms] OR "Staphylococcus aureus"[MeSH Terms] OR "Vancomycin-Resistant Staphylococcus aureus"[MeSH Terms]) AND ("Livestock"[MeSH Major Topic:noexp] OR "animals, domestic"[MeSH Terms] OR "Cattle"[MeSH Terms] OR "Poultry"[MeSH Terms] OR "Cow"[Title/Abstract] OR "Goat"[Title/Abstract] OR "Sheep"[Title/Abstract] OR "Chicken"[Title/Abstract] OR "Buffalo"[Title/Abstract] OR ("Swine"[MeSH Terms] OR "pigs*"[Title/Abstract])) AND ("Hong Kong"[Title/Abstract] OR "Hong Kong SAR"[Title/Abstract] OR "Hong Kong Special Administrative Region"[Title/Abstract])
**China**	("Methicillin-Resistant Staphylococcus aureus"[MeSH Terms] OR "Staphylococcus aureus"[MeSH Terms] OR "Vancomycin-Resistant Staphylococcus aureus"[MeSH Terms]) AND ("Livestock"[MeSH Major Topic:noexp] OR "animals, domestic"[MeSH Terms] OR "Cattle"[MeSH Terms] OR "Poultry"[MeSH Terms] OR "Cow"[Title/Abstract] OR "Goat"[Title/Abstract] OR "Sheep"[Title/Abstract] OR "Chicken"[Title/Abstract] OR "Buffalo"[Title/Abstract] OR ("Swine"[MeSH Terms] OR "pigs*"[Title/Abstract])) AND ("China"[Title/Abstract] OR "chin*"[Title/Abstract] OR "greater chin*"[Title/Abstract])
**SriLanka**	"Methicillin-Resistant Staphylococcus aureus"[MeSH Terms] OR "Staphylococcus aureus"[MeSH Terms] OR "Vancomycin-Resistant Staphylococcus aureus"[MeSH Terms]) AND ("Livestock"[MeSH Major Topic:noexp] OR "animals, domestic"[MeSH Terms] OR "Cattle"[MeSH Terms] OR "Poultry"[MeSH Terms] OR "Cow"[Title/Abstract] OR "Goat"[Title/Abstract] OR "Sheep"[Title/Abstract] OR "Chicken"[Title/Abstract] OR "Buffalo"[Title/Abstract] OR ("Swine"[MeSH Terms] OR "pig"[Title/Abstract])) AND ("Srilanka"[Title/Abstract] OR "srilank*"[Title/Abstract])
**Bangladesh**	("Methicillin-Resistant Staphylococcus aureus"[MeSH Terms] OR "Staphylococcus aureus"[MeSH Terms] OR "Vancomycin-Resistant Staphylococcus aureus"[MeSH Terms]) AND ("Livestock"[MeSH Major Topic:noexp] OR "animals, domestic"[MeSH Terms] OR "Cattle"[MeSH Terms] OR "Poultry"[MeSH Terms] OR "Cow"[Title/Abstract] OR "Goat"[Title/Abstract] OR "Sheep"[Title/Abstract] OR "Chicken"[Title/Abstract] OR "Buffalo"[Title/Abstract] OR ("Swine"[MeSH Terms] OR "pig"[Title/Abstract])) AND ("Bangladesh"[Title/Abstract] OR "bangl*"[Title/Abstract] OR "bengal*"[Title/Abstract])

Supplement Table S3: An example of search results from PubMed for different countries, including Hong Kong, China, Sri Lanka, and Bangladesh.

Search Number	Query	Search Details	Results
**12**	((((("Methicillin-Resistant Staphylococcus aureus"[MeSH Terms]) OR ("Staphylococcus aureus"[MeSH Terms])) OR ("Vancomycin-Resistant Staphylococcus aureus"[MeSH Terms])) AND (((((((((("Livestock"[Majr:NoExp]) OR "Animals, Domestic"[Mesh]) OR "Cattle"[Mesh]) OR "Poultry"[Mesh]) OR (Cow[Title/Abstract])) OR (Goat[Title/Abstract])) OR (Sheep[Title/Abstract])) OR (Chicken[Title/Abstract])) OR (Buffalo[Title/Abstract])) OR (("Swine"[Mesh]) OR (Pigs*[Title/Abstract])))) AND ((("Hong Kong"[Title/Abstract]) OR ("Hong Kong SAR"[Title/Abstract])) OR ("Hong Kong Special Administrative Region"[Title/Abstract]))	("Methicillin-Resistant Staphylococcus aureus"[MeSH Terms] OR "Staphylococcus aureus"[MeSH Terms] OR "Vancomycin-Resistant Staphylococcus aureus"[MeSH Terms]) AND ("Livestock"[MeSH Major Topic:noexp] OR "animals, domestic"[MeSH Terms] OR "Cattle"[MeSH Terms] OR "Poultry"[MeSH Terms] OR "Cow"[Title/Abstract] OR "Goat"[Title/Abstract] OR "Sheep"[Title/Abstract] OR "Chicken"[Title/Abstract] OR "Buffalo"[Title/Abstract] OR ("Swine"[MeSH Terms] OR "pigs*"[Title/Abstract])) AND ("Hong Kong"[Title/Abstract] OR "Hong Kong SAR"[Title/Abstract] OR "Hong Kong Special Administrative Region"[Title/Abstract])	7
**11**	((((("Methicillin-Resistant Staphylococcus aureus"[MeSH Terms]) OR ("Staphylococcus aureus"[MeSH Terms])) OR ("Vancomycin-Resistant Staphylococcus aureus"[MeSH Terms])) AND (((((((((("Livestock"[Majr:NoExp]) OR "Animals, Domestic"[Mesh]) OR "Cattle"[Mesh]) OR "Poultry"[Mesh]) OR (Cow[Title/Abstract])) OR (Goat[Title/Abstract])) OR (Sheep[Title/Abstract])) OR (Chicken[Title/Abstract])) OR (Buffalo[Title/Abstract])) OR (("Swine"[Mesh]) OR (Pigs*[Title/Abstract])))) AND (((China[Title/Abstract]) OR (Chin*[Title/Abstract])) OR ("Greater Chin*"[Title/Abstract]))	("Methicillin-Resistant Staphylococcus aureus"[MeSH Terms] OR "Staphylococcus aureus"[MeSH Terms] OR "Vancomycin-Resistant Staphylococcus aureus"[MeSH Terms]) AND ("Livestock"[MeSH Major Topic:noexp] OR "animals, domestic"[MeSH Terms] OR "Cattle"[MeSH Terms] OR "Poultry"[MeSH Terms] OR "Cow"[Title/Abstract] OR "Goat"[Title/Abstract] OR "Sheep"[Title/Abstract] OR "Chicken"[Title/Abstract] OR "Buffalo"[Title/Abstract] OR ("Swine"[MeSH Terms] OR "pigs*"[Title/Abstract])) AND ("China"[Title/Abstract] OR "chin*"[Title/Abstract] OR "greater chin*"[Title/Abstract])	135

**10**	((((("Methicillin-Resistant Staphylococcus aureus"[MeSH Terms]) OR ("Staphylococcus aureus"[MeSH Terms])) OR ("Vancomycin-Resistant Staphylococcus aureus"[MeSH Terms])) AND ((((((((("Livestock"[Majr:NoExp]) OR "Animals, Domestic"[Mesh]) OR "Cattle"[Mesh]) OR "Poultry"[Mesh]) OR (Cow[Title/Abstract])) OR (Goat[Title/Abstract])) OR (Sheep[Title/Abstract])) OR (Chicken[Title/Abstract])) OR (Buffalo[Title/Abstract])) OR (("Swine"[Mesh]) OR (Pig*[Title/Abstract])))) AND ((Srilanka[Title/Abstract]) OR (SriLank*[Title/Abstract]))	("Methicillin-Resistant Staphylococcus aureus"[MeSH Terms] OR "Staphylococcus aureus"[MeSH Terms] OR "Vancomycin-Resistant Staphylococcus aureus"[MeSH Terms]) AND ("Livestock"[MeSH Major Topic:noexp] OR "animals, domestic"[MeSH Terms] OR "Cattle"[MeSH Terms] OR "Poultry"[MeSH Terms] OR "Cow"[Title/Abstract] OR "Goat"[Title/Abstract] OR "Sheep"[Title/Abstract] OR "Chicken"[Title/Abstract] OR "Buffalo"[Title/Abstract] OR ("Swine"[MeSH Terms] OR "pig"[Title/Abstract])) AND ("Srilanka"[Title/Abstract] OR "srilank*"[Title/Abstract])	0
**9**	((((("Methicillin-Resistant Staphylococcus aureus"[MeSH Terms]) OR ("Staphylococcus aureus"[MeSH Terms])) OR ("Vancomycin-Resistant Staphylococcus aureus"[MeSH Terms])) AND ((((((((("Livestock"[Majr:NoExp]) OR "Animals, Domestic"[Mesh]) OR "Cattle"[Mesh]) OR "Poultry"[Mesh]) OR (Cow[Title/Abstract])) OR (Goat[Title/Abstract])) OR (Sheep[Title/Abstract])) OR (Chicken[Title/Abstract])) OR (Buffalo[Title/Abstract])) OR (("Swine"[Mesh]) OR (Pig*[Title/Abstract])))) AND (((Bangladesh[Title/Abstract]) OR (Bangl*[Title/Abstract])) OR (Bengal*[Title/Abstract]))	("Methicillin-Resistant Staphylococcus aureus"[MeSH Terms] OR "Staphylococcus aureus"[MeSH Terms] OR "Vancomycin-Resistant Staphylococcus aureus"[MeSH Terms]) AND ("Livestock"[MeSH Major Topic:noexp] OR "animals, domestic"[MeSH Terms] OR "Cattle"[MeSH Terms] OR "Poultry"[MeSH Terms] OR "Cow"[Title/Abstract] OR "Goat"[Title/Abstract] OR "Sheep"[Title/Abstract] OR "Chicken"[Title/Abstract] OR "Buffalo"[Title/Abstract] OR ("Swine"[MeSH Terms] OR "pig"[Title/Abstract])) AND ("Bangladesh"[Title/Abstract] OR "bangl*"[Title/Abstract] OR "bengal*"[Title/Abstract])	8
**8**	(("Hong Kong"[Title/Abstract]) OR ("Hong Kong SAR"[Title/Abstract])) OR ("Hong Kong Special Administrative Region"[Title/Abstract])	"Hong Kong"[Title/Abstract] OR "Hong Kong SAR"[Title/Abstract] OR "Hong Kong Special Administrative Region"[Title/Abstract]	24,069
**7**	((China[Title/Abstract]) OR (Chin*[Title/Abstract])) OR ("Greater Chin*"[Title/Abstract])	"China"[Title/Abstract] OR "chin*"[Title/Abstract] OR "greater chin*"[Title/Abstract]	521,492

**6**	(Srilanka[Title/Abstract]) OR (SriLank*[Title/Abstract])	"Srilanka"[Title/Abstract] OR "srilank*"[Title/Abstract]	106
**5**	((Bangladesh[Title/Abstract]) OR (Bangl*[Title/Abstract])) OR (Bengal*[Title/Abstract])	"Bangladesh"[Title/Abstract] OR "bangl*"[Title/Abstract] OR "bengal*"[Title/Abstract]	31,513
**4**	((((((((("Livestock"[Majr:NoExp]) OR "Animals, Domestic"[Mesh]) OR "Cattle"[Mesh]) OR "Poultry"[Mesh]) OR (Cow[Title/Abstract])) OR (Goat[Title/Abstract])) OR (Sheep[Title/Abstract])) OR (Chicken[Title/Abstract])) OR (Buffalo[Title/Abstract])) OR (("Swine"[Mesh]) OR (Pig*[Title/Abstract]))	"Livestock"[MeSH Major Topic:noexp] OR "animals, domestic"[MeSH Terms] OR "Cattle"[MeSH Terms] OR "Poultry"[MeSH Terms] OR "Cow"[Title/Abstract] OR "Goat"[Title/Abstract] OR "Sheep"[Title/Abstract] OR "Chicken"[Title/Abstract] OR "Buffalo"[Title/Abstract] OR "Swine"[MeSH Terms] OR "pig"[Title/Abstract]	
**3**	(((((((("Livestock"[Majr:NoExp]) OR "Animals, Domestic"[Mesh]) OR "Cattle"[Mesh]) OR "Poultry"[Mesh]) OR (Cow[Title/Abstract])) OR (Goat[Title/Abstract])) OR (Sheep[Title/Abstract])) OR (Chicken[Title/Abstract])) OR (Buffalo[Title/Abstract])	"Livestock"[MeSH Major Topic:noexp] OR "animals, domestic"[MeSH Terms] OR "Cattle"[MeSH Terms] OR "Poultry"[MeSH Terms] OR "Cow"[Title/Abstract] OR "Goat"[Title/Abstract] OR "Sheep"[Title/Abstract] OR "Chicken"[Title/Abstract] OR "Buffalo"[Title/Abstract]	698,827
**2**	("Swine"[Mesh]) OR (Pig*[Title/Abstract])	"Swine"[MeSH Terms] OR "pig"[Title/Abstract]	325,258
**1**	((("Methicillin-Resistant Staphylococcus aureus"[MeSH Terms]) OR ("Staphylococcus aureus"[MeSH Terms])) OR ("Vancomycin-Resistant Staphylococcus aureus"[MeSH Terms])	"Methicillin-Resistant Staphylococcus aureus"[MeSH Terms] OR "Staphylococcus aureus"[MeSH Terms] OR "Vancomycin-Resistant Staphylococcus aureus"[MeSH Terms]	88,128

Supplementary Table S4: Study inclusion and exclusion criteria.

PEO framework	Inclusion criteria	Exclusion criteria
**Population/subjects**	This systematic review will be conducted on animals. Therefore, we consider animals as a population. We will include studies conducted on animals, including livestock, cattle, poultry, cows, goats, sheep, chicken buffalo, swine, pigs, and companion animals.<br>Studies will have to have >30 subjects.	Studies conducted on subjects other than the animals mentioned in the inclusion criteria will be excluded.<br>Studies involving subjects less than 30 will be excluded.
**Exposure**	*S. aureus*, Methicillin resistant *S. aureus* (MRSA).	Subjects exposed to bacteria other than this will be excluded.
**Outcomes**	Prevalence or percentage of *S. aureus* or MRSA among subjects.	Studies that do not have clear data about the prevalence of MRSA will be excluded.
**Other criteria**		
**Language**	Studies conducted in English and Chinese language.	Other than English and Chinese language.
**Publication year**	We have limited studies in some countries, so we did not have any previous publication date as inclusion criteria.	Our data collection will be ended by January 2024. Thus, studies published beyond this period will be excluded.
**Study design**	Observational study, including cross-sectional, longitudinal, or cohort study, will be included.	All types of review articles, editorials, news, letters, short communications, and grey literature will be excluded.
**Types of paper**	Full-text articles	Unpublished or any conference proceedings.

Supplementary Table S5: Data collection form

Criteria	Information
**Author names and publication year**	
**Country or region**	
**Study period**	
**Study design**	
**Screening site(s)**	
**Types of livestock or animals**	
**Farm criteria (big or small)**	
**Place of farm (city or rural)**	
**Total number of target subjects**	
**Clinical signs and symptoms**	
**MRSA identification method**	
**S. aureus screening method**	
**Pre-enrichment before detection (yes/no)**	
**Primary outcomes**	
**S. aureus prevalence (positive/total)**	
**MRSA prevalence (positive/total)**	
**Secondary outcomes**	
**Significant risk factors for MRSA carriage**	
**Antibiotic resistance profile of MRSA**	
**Molecular genetic analysis of MRSA (ST/CC)**	
**Hygienic condition of the farm**	

Hygienic practices

Clinical signs and symptoms

Supplementary Table S6: Characteristics of the studies

Study ID	MRSA	SA	Total Sample	Sampling												Country or region	Location	Pre-enrichment	MRSA detection method	Study type
				Livestock	Livestock-MRSA	Livestock-SA	Poultry	Poultry-MRSA	Poultry-SA	Raw food products	Raw food products - MRSA	Raw food products -SA	Pets	Pets-MRSA	Pets-SA					
Ain 2022	6	8	60	ND	ND	ND	ND	ND	ND	60	6	8	ND	ND	ND	Bangladesh	Urban	Yes	*mec*A and/or *mec*C PCR	Cross-sectional study
Ali 2016	12	54	60	60	12	54	ND	ND	ND	ND	ND	ND	ND	ND	ND	Bangladesh	Urban	Yes	*mec*A and/or *mec*C PCR	Cross-sectional study
Chang 2016	4	336	3476	ND	ND	ND	ND	ND	ND	3476	4	336	ND	ND	ND	China	Urban	Yes	*mec*A and/or *mec*C PCR	Cross-sectional study
Chen 2021	9	125	466	ND	ND	ND	ND	ND	ND	466	9	125	ND	ND	ND	China	Not mentioned	Yes	Broth Microdilution	Cross-sectional study
Cui 2022	27	68	139	139	27	68	ND	ND	ND	ND	ND	ND	ND	ND	ND	China	Urban	Yes	*mec*A and/or *mec*C PCR	Cross-sectional study
Fan 2015	0	86	265				ND	ND	ND	265	0	86	ND	ND	ND	China	Urban	Yes	Broth Microdilution	Cross-sectional study
Fazal 2023	28	98	284	ND	ND	ND	ND	ND	ND	284	28	98	ND	ND	ND	Bangladesh	Rural	Yes	*mec*A and/or *mec*C PCR	Cross-sectional study
Gan 2021	24	236	3584	183	1	15	3401	23	221	ND	ND	ND	ND	ND	ND	China	Not mentioned	Yes	Broth Microdilution	Cross-sectional study

Hao 2015	7	56	94	ND	ND	ND	ND	ND	ND	94	7	65	ND	ND	ND	China	Urban	Yes	Disk Diffusion	Cross-sectional study
Haque 2018	14	57	72	72	14	57	ND	ND	ND	72	14	57	ND	ND	ND	Bangladesh	Urban	Yes	*mec*A and/or *mec*C PCR	Cross-sectional study
He 2013	20	49	250	160	20	16	ND	ND	ND	90	0	13	ND	ND	ND	China	Urban	Yes	Agar Dilution	Cross-sectional study
Hoque 2018	81	145	175	175	81	145	ND	ND	ND	ND	ND	ND	ND	ND	ND	Bangladesh	Rural	Yes	*mec*A and/or *mec*C PCR	Cross-sectional study
Hoque 2022	29	109	500	500	29	109	ND	ND	ND	ND	ND	ND	ND	ND	ND	Bangladesh	Rural	Yes	Disk Diffusion	Cross-sectional study
Hossain 2022	45	57	100	ND	ND	ND	100	45	57	ND	ND	ND	ND	ND	ND	Bangladesh	Urban	Yes	*mec*A and/or *mec*C PCR	Cross-sectional study
Hu 2020	5	63	110	ND	ND	ND	110	5	63	ND	ND	ND	ND	ND	ND	China	Urban	Yes	*mec*A and/or *mec*C PCR	Cross-sectional study
Islam 2011	0	54	100	100	0	54	ND	ND	ND	ND	ND	ND	ND	ND	ND	Bangladesh	Not mentioned	Yes	*mec*A and/or *mec*C PCR	Cross-sectional study
Jahan 2014	0	12	47	ND	ND	ND	ND	ND	ND	47	0	12	ND	ND	ND	Bangladesh	Urban	Yes	*mec*A and/or *mec*C PCR	Cross-sectional study
Jayaweera 2017	26	72	188	124	20	32	ND	ND	ND	64	6	20	ND	ND	ND	Sri Lanka	Not mentioned	Yes	not mentioned	Cross-sectional study

Kalupahana 2019	6	6	493	493	6	6	ND	ND	ND	ND	ND	ND	ND	ND	ND	Sri Lanka	Urban	Yes	Disk Diffusion	Cross-sectional study
Kou 2021	32	62	144	ND	ND	ND	ND	ND	ND	144	32	62	ND	ND	ND	China	Rural	Yes	*mec*A and/or *mec*C PCR	Cross-sectional study
Li 2009	0	106	846	ND	ND	ND	ND	ND	ND	846	0	106	ND	ND	ND	China	Rural	Not mentioned	Disk Diffusion	Cross-sectional study
Li 2015	1	121	214	ND	ND	ND	ND	ND	ND	214	1	121	ND	ND	ND	China	Urban	Yes	Selective agar plate	Cross-sectional study
Li 2017	89	174	348	348	174	89	ND	ND	ND	ND	ND	ND	ND	ND	ND	China	Urban	Not mentioned	not mentioned	Cross-sectional study
Li 2019a	101	480	6500	ND	ND	ND	6500	101	480	ND	ND	ND	ND	ND	ND	China	Rural	Yes	*mec*A and/or *mec*C PCR	Cross-sectional study
Li 2019b	8	104	507	ND	ND	ND	507	8	104	ND	ND	ND	ND	ND	ND	China	Not mentioned	Not mentioned	Disk Diffusion	Cross-sectional study
Li 2022a	18	62	1667	580	7	20	698	3	17	389	8	25	ND	ND	ND	China	Rural	Yes	*mec*A and/or *mec*C PCR	Cross-sectional study
Li 2022b	85	199	585	585	85	199	ND	ND	ND	ND	ND	ND	ND	ND	ND	China	Urban	Yes	Disk Diffusion	Cross-sectional study
Liu 2017	16	54	195	ND	ND	ND	ND	ND	ND	195	16	54	ND	ND	ND	China	Urban	Yes	Disk Diffusion	Cross-sectional study

Liu 2020	8	62	624	ND	ND	ND	ND	ND	ND	624	62	8	ND	ND	ND	China	Not mentioned	No	*mec*A and/or *mec*C PCR	Cross-sectional study
Liu 2021	26	128	1324	1324	26	128	ND	ND	ND	ND	ND	ND	ND	ND	ND	China	Urban	Yes	*mec*A and/or *mec*C PCR	Cross-sectional study
Liu 2022	6	36	125	ND	ND	ND	ND	ND	ND	125	6	36	ND	ND	ND	China	Not mentioned	No	Disk Diffusion	Cross-sectional study
Madurangi 2013	0	32	40	ND	ND	ND	ND	ND	ND	40	0	32	ND	ND	ND	Sri Lanka	Urban	Yes	*mec*A and/or *mec*C PCR	Cross-sectional study
Memon 2012	9	34	714	ND	ND	ND	ND	ND	ND	714	9	34	ND	ND	ND	China	Not mentioned	Not mentioned	Broth Microdilution	Cross-sectional study
Parvin 2021	23	62	113	ND	ND	ND	113	23	62	ND	ND	ND	ND	ND	ND	Bangladesh	Urban	Yes	Disk Diffusion	Cross-sectional study
Parvin 2022	23	49	50	ND	ND	ND	50	23	49	ND	ND	ND	ND	ND	ND	Bangladesh	Urban	Yes	*mec*A and/or *mec*C PCR	Cross-sectional study
Rafiq 2022	81	404	2320	ND	ND	ND	2320	8	404	ND	ND	ND	ND	ND	ND	Bangladesh	Urban	Yes	*mec*A and/or *mec*C PCR	Cross-sectional study
Rahman 2005	4	54	100	100	4	54	ND	ND	ND	ND	ND	ND	ND	ND	ND	Bangladesh	Not mentioned	n	*mec*A and/or *mec*C PCR	Cross-sectional study
Rahman 2018	0	18	100	55	0	18	ND	ND	ND	ND	ND	ND	45	4	11	Bangladesh	Urban	Yes	*mec*A and/or *mec*C PCR	Cross-sectional study

Rana 2020	6	49	67	67	6	49	ND	ND	ND	ND	ND	ND	ND	ND	ND	Bangladesh	Rural	Yes	*mec*A and/or *mec*C PCR	Cross-sectional study
Re 2020	0	65	84	ND	ND	ND	ND	ND	ND	84	65	0	ND	ND	ND	China	Urban	Yes	Disk Diffusion	Cross-sectional study
Salauddin 2020	48	48	448	448	48	48	ND	ND	ND	ND	ND	ND	ND	ND	ND	Bangladesh	Urban	Not mentioned	Disk Diffusion	Cross-sectional study
Shi 2021	174	276	750	ND	ND	ND	ND	ND	ND	750	174	276	ND	ND	ND	China	Not mentioned	Yes	Disk Diffusion	Cross-sectional study
Sumon 2018	12	20	104	104	12	20	ND	ND	ND	ND	ND	ND	ND	ND	ND	Bangladesh	Rural	Yes	Disk Diffusion	Cross-sectional study
Sun 2019	120	221	829	815	ND	209	ND	ND	ND	14	2	2	ND	ND	ND	China	Rural	Yes	Selective agar plate	Cross-sectional study
Tang 2022	0	45	142	55	0	23	ND	ND	ND	87	0	22	ND	ND	ND	China	Not mentioned	Not mentioned	Broth Microdilution	Cross-sectional study
Wang 2012	100	14	332	234	14	96	ND	ND	ND	98	0	30	ND	ND	ND	China	Not mentioned	Yes	Agar Dilution	Cross-sectional study
Wang 2013	20	279	1152	ND	ND	ND	ND	ND	ND	1152	20	279	ND	ND	ND	China	Urban	Yes	*mec*A and/or *mec*C PCR	Cross-sectional study
Wang 2014	206	11	719	160	1	55	264	6	108	295	4	43	ND	ND	ND	China	Urban	Yes	Disk Diffusion	Cross-sectional study

Wang 2018a	1	96	195	ND	ND	ND	ND	ND	ND	195	1	96	ND	ND	ND	China	Urban	Yes	*mec*A and/or *mec*C PCR	Cross-sectional study
Wang 2018b	0	53	464	ND	ND	ND	ND	ND	ND	464	0	53	ND	ND	ND	China	Urban	Yes	*mec*A and/or *mec*C PCR	Cross-sectional study
Wu 2019	107	1581	4300	ND	ND	ND	ND	ND	ND	4300	107	1581	ND	ND	ND	China	Urban	Not mentioned	Disk Diffusion	Cross-sectional study
Xu 2015	10	28	209	ND	ND	ND	ND	ND	ND	209	10	28	ND	ND	ND	China	Urban	No	Disk Diffusion	Cross-sectional study
Xu 2021	21	230	814	814	21	230	ND	ND	ND	ND	ND	ND	ND	ND	ND	China	Urban	Yes	Broth Microdilution	Cross-sectional study
Yan 2014	0	200	590	590	0	200	ND	ND	ND	ND	ND	ND	ND	ND	ND	China	Urban	Yes	*mec*A and/or *mec*C PCR	Cross-sectional study
Yang 2016	7	47	399	ND	ND	ND	ND	ND	ND	399	7	47	ND	ND	ND	China	Urban	Yes	Disk Diffusion	Cross-sectional study
Yang 2020	73	498	3136	ND	ND	ND	ND	ND	ND	3136	73	498	ND	ND	ND	China	Not mentioned	Not mentioned	Disk Diffusion	Cross-sectional study
Ye 2020	35	43	426	426	35	43	ND	ND	ND	ND	ND	ND	ND	ND	ND	China	Urban	Yes	Disk Diffusion	Cross-sectional study
Zeng 2018	104	141	2997	ND	ND	ND	ND	ND	ND	2997	104	141	ND	ND	ND	China	Urban	Not mentioned	*mec*A and/or *mec*C PCR	Cross-sectional study

Zhang 2011	21	397	2745	ND	ND	ND	ND	ND	ND	ND	ND	ND	2745	21	397	China	Urban	Yes	Broth Microdilution	Cross-sectional study
Zhang 2016	11	58	200	ND	ND	ND	ND	ND	ND	200	11	58	ND	ND	ND	China	Not mentioned	No	Disk Diffusion	Cross-sectional study
Zhang 2018	0	103	1021	ND	ND	ND	ND	ND	ND	1021	0	103	ND	ND	ND	China	Urban	Yes	Broth Microdilution	Cross-sectional study
Zhang 2020a	7	163	590	590	7	163	ND	ND	ND	ND	ND	ND	ND	ND	ND	China	Not mentioned	Not mentioned	Disk Diffusion	Cross-sectional study
Zhang 2020b	5	27	218	ND	ND	ND	ND	ND	ND	218	5	27	ND	ND	ND	China	Urban	Yes	Broth Microdilution	Cross-sectional study
Zhang 2020c	34	462	56	ND	ND	ND	ND	ND	ND	462	34	56	ND	ND	ND	China	Not mentioned	Not mentioned	Broth Microdilution	Cross-sectional study
Zhang 2022	298	73	2962	ND	ND	ND	ND	ND	ND	2962	73	298	ND	ND	ND	China	Not mentioned	No	*mec*A and/or *mec*C PCR	Cross-sectional study
Zhou 2017	0	32	74	74	0	32	ND	ND	ND	ND	ND	ND	ND	ND	ND	China	Urban	Yes	Disk Diffusion	Cross-sectional study
Zhou 2020	22	149	595	338	12	90	ND	ND	ND	160	10	37	ND	ND	ND	China	Not mentioned	Yes	Broth Microdilution	Cross-sectional study
Zhu 2022	19	518	3067	ND	ND	ND	ND	ND	ND	3027	19	518	ND	ND	ND	China	Urban	Yes	Broth Microdilution	Cross-sectional study

Zou 2022	7	83	1291	1291	7	83	ND	ND	ND	ND	ND	ND	ND	ND	ND	China	Rural	Yes	Broth Microdilution	Cross-sectional study

Supplementary Table S7: Publication bias summary

**Egger's test for SA**	
Intercept	9.18
95% CI	5.64 to 12.72
Significance level	$p < 0.0001$
**Begg's test for SA**	
Kendall's Tau	0.36
Significance level	$p < 0.0001$
**Egger's test for MRSA**	
**Intercept**	4.90
95% CI	2.43 to 7.37
Significance level	$p = 0.0002$
**Begg's test for MRSA**	
Kendall's Tau	0.23
Significance level	$p = 0.0044$

Supplementary Table S8:Antibiotic resistance characterisation

Studies	Antibiotics	No. of Studies	No. of Isolates	Pooled Prevalence	I^2(%)
**MACROLIDES**					
Zhang 2011	**Azithromycin**	1	21	---	---
Chen 2021	**Erythromycin**	16	9	81.28%(64.47%- 93.57)	96.21%
Cui 2022			27		
Gan 2021			24		
Kalupahana 2019			3		
Li 2022a			15		
Liu 2021			13		
Li 2019b			6		
Sun 2019			116		
Wang 2012			14		
Wang 2014			197		
Wang 2018a			1		
Wu 2019			89		
Yang 2016			5		
Ye 2020			35		
Zeng 2018			102		
Zou 2022			1		
Wang 2014	**Midecamycin**	1	152	---	---
**TETRACYCLINES**					
Ain 2022	**Doxycycline**	2	5	61.9% (24.6% - 92.4%)	50.30%
Hu 2020			2		
Wang 2014	**Clarithromycin**	1	179	---	---
Li 2019a	**Tetracycline**	13	97	72.7% (54.5% - 87.5%)	96.88%
Li 2022a			15		
Liu 2021			19		
Li 2019b			2		
Sun 2019			97		
Wang 2012			14		
Wang 2014			134		
Wu 2019			70		
Yang 2016			5		
Yang 2020			72		
Ye 2020			35		
Zeng 2018			100		
Zhang 2011			4		
Parvin 2021	**Oxytetracycline**	1	18	---	---
**QUINOLONES**					
Wang 2014	**Ofloxacin**	1	45	---	---
Wang 2018a	**Enrofloxacin**	1	1	---	---
Li 2022a	**Levofloxacin**	2	13	60.1% (47.034% - 72.5%)	40.00%
Wang 2014			116		
Wang 2014	**Norfloxacin**	2	99	37.1% (17.6% - 59.1%)	93.14%
Wu 2019			28		
Jayaweera 2017	**Ciprofloxacin**	8	26	68.2% (42.6% to 89.0%)	97.63%
Li 2022a			14		
Sun 2019			97		
Wang 2012			14		
Wang 2014			108		
Wang 2018a			1		
Wu 2019			30		
Zeng 2018			98		
**PLEUROMUTILIN**					
Li 2022a	**Valnemulin**	2	3	56% (0.5% to 99.8%)	97.52%
Zeng 2018			93		
**CEPHALOSPORINS**					
Wang 2014	**Cefuroxime**	1	72	---	---
Wang 2014	**Cefalotin**	1	18	---	---
Wang 2014	**Cefazolin**	1	125	---	---
Chen 2021	**Ceftiofur**	2	9	97.2% (84.8% - 99.6%)	0.00%
Zou 2022			7		
Ain 2022	**Cefixime**	2	6	84.4% (54.0 % - 99.6%)	63.03%
Parvin 2021			17		
Li 2019a	**Cefotaxime**	1	97	---	---

Wang 2014	**Ceftazidime**	2	81	73.8% (10.9% - 97.5%)	99.31%
Wu 2019			104		
	**STREPTOGRAMIN**				
Li 2022a	**quinupristin/dalfopristin**	2	3	8.8% (0.5% - 25.5%)	72.55%
Wu 2019			4		
	**AMINOGLYCOSIDES**				
Li 2022a			13		
Wang 2014	**Amikacin**	3	81	41.3% (22.8% - 61.2%)	90.07%
Wu 2019			24		
Chen 2021	**Gentamicin**	10	9		
Jayaweera 2017			26		
Li 2019a			95		
Li 2022a			13		
Sun 2019			97	78.3% (58.6% - 92.8%)	97.11%
Wang 2014			108		
Wu 2019			30		
Yang 2020			73		
Zeng 2018			92		
Zhang 2011			4		
Li 2019b			2		
Kalupahana 2019			1		
Wang 2014			152		
Wu 2019	**Kanamycin**	5	81	61.70%(46.46%- 75.86%)	76.05%
Yang 2016			5		
Wang 2014			134		
Wu 2019	**Streptomycin**	4	72	66.9% (53.5% - 79.2%)	71.38%
Zhang 2020a			7		
Zou 2022			2		
Wang 2014	**Tobramycin**	1	116	---	---
	**MISCELLANEOUS**				
Chen 2021			9		
Cui 2022			27		
Jayaweera 2017			26		
Li 2022a			15		
Li 2019b			1		
Sun 2019	**Clindamycin**	11	116	91.7% (82.8% - 97.5%)	90.04%
Wang 2014			206		
Wu 2019			85		
Yang 2016			5		
Zeng 2018			100		
Zhang 2011			21		
Li 2022a			8		
Wu 2019	**Rifampin**	3	8	12.7%(1.7% to 31.8%)	90.90%
Zeng 2018			2		
	**TRIMETHOPRIM-SULFAMETHOXAZOLE**				
Li 2022a			6		
Wang 2012			14		
Wang 2014			143		
Wu 2019	**Trimethoprim-sulfamethoxazole**	7	14	61.6% (31.0% - 87.8%)	97.97%
Xu 2021			21		
Zeng 2018			92		
Zhang 2020b			5		
**GLYCOPEPTIDES**					
Liu 2021	**Vancomycin**	1	15	---	---
Wu 2019	**Teicoplanin**	1	1	---	---
**FUSIDANE**					
Wu 2019	Fusidic Acid	1	30	---	---
**NITROFURAN**					
Wang 2014	**Nitrofurantoin**	2	27	7.6%(0.8% - 20.4%)	90.50%
Wu 2019			3		
**BETA LACTAMS**					
Ain 2022			6		
Chen 2021			9		
Gan 2021			24		
Li 2022a			18		
Parvin 2021	**Ampicillin**	9	22	99.6%(98.9% - 99.9%)	0.00%
Wang 2014			206		
Wu 2019			107		
Yang 2016			7		
Zeng 2018			104		

Gan 2021			24		
Li 2022a			18		
Liu 2017			16		
Liu 2021			26		
Parvin 2021			19		
Li 2019b			8		
Wang 2014			206		
Wang 2018a	**Penicillin**	16	1	98.8%(97.2% - 99.7%)	41.92%
Wu 2019			107		
Yang 2016			7		
Yang 2020			73		
Ye 2020			35		
Zeng 2018			104		
Zhang 2011			21		
Zhu 2022			19		
Zou 2022			5		
Chang 2016			4		
Gan 2021			24		
Li 2015			1		
Li 2022a			18		
Liu 2017			16		
Liu 2022			8		
Parvin 2021			23		
Li 2019b			8		
Sun 2019	**Cefoxitin**	17	120	92.7% (73.7% - 99.9%)	98.45%
Wang 2012			14		
Wang 2014			206		
Xu 2015			9		
Yang 2020			73		
Zeng 2018			104		
Zhang 2020a			7		
Zhang 2020b			5		
Zhang 2022			73		
Ain 2022			6		
Parvin 2021	**Amoxicillin**	3	20	87.00% (80.9% - 92.1%)	0.00%
Wu 2019			93		
Parvin 2021	**Amoxicillin-clavunic acid**	1	20	---	---
Ain 2022			5		
Chen 2021			9		
Gan 2021			24		
Hossain 2022			45		
Kou 2021			32		
Li 2015			1		
Li 2022a			15		
Liu 2020			8		
Liu 2022			6		
Parvin 2021			22		
Parvin 2022	**Oxacilllin**	**21**	32	98.8%(97.8% - 99.6%)	19.25%
Rafiq 2022			81		
Shi 2021			174		
Wang 2014			206		
Xu 2021			21		
Zeng 2018			102		
Zhang 2011			21		
Zhang 2020a			7		
Zhou 2020			22		
Zhu 2022			19		
Zou 2022			7		
Cui 2022	**Topenicillin**	**1**	27	**---**	**---**
Wang 2014	**Cefepime**	2	81	52.2% (27.4% - 76.5%)	94.88%
Wu 2019			70		
Parvin 2021	**Cloxacillin**	1	19	---	---
**CHLORAMPHENICOL**					
Wang 2014	**Ceftriaxone**	2	63	72.6% (2.6% - 90.8%)	98.36%
Zhang 2011			21		
Wang 2012			14		
Wu 2019	**Chloramphenicol**	3	40	16.0% (2.1% - 39.4%)	95.15%
Yang 2020			2		
Wang 2014	**Chloromycetin**	1	108	---	---
Sulfonamide					
Gan 2021	**Sulfafurazole**	1	24	---	---
**POLYCATIONIC PEPTIDE**					
Parvin 2021	**Colistin**	1	21	---	---

**KETOLIDES**					
Wu 2019	**Telithromycin**	1	79	---	---
**AMINOCYCLITOL**					
Wang 2014	**Spectinomycin**	1	152	---	---
**PHOSPHONIC**					
Hu 2020	**Fosfomycin**	1	5	---	---
